# Solubility Challenges and Strategies for Organic Sodium‐Ion Batteries: Status and Perspectives

**DOI:** 10.1002/smll.202412769

**Published:** 2025-02-24

**Authors:** Ying Qi, Huaping Zhao, Lizhuang Chen, Yong Lei

**Affiliations:** ^1^ School of Environmental and Chemical Engineering Jiangsu University of Science and Technology Zhenjiang Jiangsu 212003 P. R. China; ^2^ Fachgebiet Angewandte Nanophysik Institut für Physik and IMN MacroNano Technische Universität Ilmenau 98693 Ilmenau Germany

**Keywords:** electrolyte, insolubility, organic materials, separator, sodium‐ion batteries

## Abstract

Organic sodium‐ion batteries (OSIBs) possessing excellent characteristics of low price, abundant sources, and eco‐compatibility have gained numerous attentions in the recent decade. However, solubility is one of the main severe limitations of the application of OSIBs, especially for small organic molecules. The dissolution of molecules into electrolytes can cause the loss of active materials, the pulverization of electrodes, and even short circuits in batteries, as active materials may shuttle through separators, thus leading to poor cycling stability for sodium‐ion batteries. Thus, there is an urgent need to develop insoluble organic molecules for OSIBs. The advanced development of OSIBs over the past decades is overviewed, and the primary challenges faced by long‐cycling OSIBs in terms of solubility are systematically analyzed. Focusing on the three core components of the battery system electrodes, separators, and electrolytes, targeted optimization strategies are proposed to mitigate solubility issues and enhance battery performance. In addition, the prospects of OSIBs toward long‐cycle stability and practical application are explored.

## Graphical abstract

Organic sodium‐ion batteries (OSIBs) have garnered interest due to their low cost, abundant resources, and sustainability. However, the solubility of organic materials in aprotic electrolytes results in electrode deterioration, active material loss, and poor cycling stability. Here three strategies including insoluble electrode design, concentrated electrolyte optimization, and functional separator modulation are summarized for improving long‐cycling stability during the charge and discharge processes.

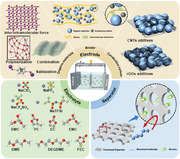

## 1 Introduction

Rechargeable batteries have evolved rapidly, driven by the boost requirements of global energy consumption and the growing focus on achieving climate neutrality. The development trajectory began with lead‐acid batteries and progressed through nickel‐based batteries to modern lithium‐ion batteries (LIBs).^[^ ^1^ ^]^ Over the past few decades, LIBs have become a cornerstone of the commercial market, due to the high energy density (≈300 Wh kg^−1^), high power density, and long cycling lifetime.^[^ ^2^, ^3^ ^]^ According to the International Energy Agency (IEA), the demand for batteries in vehicles and stationary storage is expected to grow nearly fivefold by 2030 (≈4 TWh per year) compared to 2023.^[^ ^4^ ^]^ Despite their widespread success, LIBs face significant challenges. The price of lithium tends to fluctuate drastically due to the limited and unevenly distributed lithium resources and uncertainty from geopolitical anxieties.^[^ ^5^ ^]^ These factors pose a risk to the uncontrolled supply chain for grid‐scale applications. Here, there is an urgent need to explore affordable and consistently available alternatives for practical energy storage devices. Sodium‐ion batteries (SIBs) are considered a promising complementary to LIBs due to the abundant sodium resources, lower price, and high safety.^[^ ^6^ ^]^ SIBs have experienced rapid development over the past 5 years, primarily due to their physical and chemical similarities to LIBs. This compatibility allows SIBs to leverage established scientific theories and even utilize existing production lines, accelerating their advancement.

Currently, SIBs rely on inorganic materials derived from depletable mineral resources, commonly using Prussian blue analogs as cathodes and hard carbon as anodes for commercial full cells.^[^ ^7^ ^]^ The production of these materials unavoidably leads to energy consumption and carbon emissions, exacerbating cost and environmental challenges to cope with the rapid growing market. Therefore, it is essential to develop sustainable and benign electrode materials for SIBs. Different from inorganic materials, organic materials show higher abundance, and lower cost (such as 3,4,9,10‐perylenetetracarboxylic dianhydride (PTCDA): 0.1 $ kg^−1^ vs NaNi_0.33_Fe_0.33_Mn_0.33_O_2_: 6.42 $ kg^−1^, data from Amitychem factory and Shanghai metal market), which have a potential for large‐scale application. Their natural sustainability, structural tunability, and favorable processability offer significant advantages over other inorganic materials in terms of production and recycling. In addition, the excellent flexibility and lightweight make organic SIBs (OSIBs) suitable for smart wearable devices, creating great potential for future applications.

**Figure 1:**
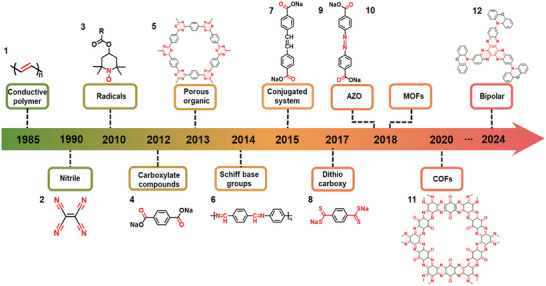
The development process of organic materials for sodium‐ion batteries with corresponding findings until 2024 (active sites highlight in red).

The investigation of organic materials for SIBs can date back to the 1980s. Inspired by advancements in LIBs,^[^ ^8^ ^]^ conductive polymers polyacetylene (PA, 1) were explored as anode materials for SIBs (**Figure** **1**).^[^ ^9^ ^]^ In 1990, tetracyanoethylene (TCNE, 2), featuring nitrile functional groups, was employed as cathode in high‐temperature semisolid SIBs, delivering a high energy density.^[^ ^10^ ^]^ After these in the next decades, SIBs remained overshadowed by the prominence of LIBs, due to the high energy and high power density of LIBs. It was not until the 2010s that development of research on SIBs began to accelerate, including studies focusing on organic materials.^[^ ^7^ ^]^ Active radical polymer 3 as a cathode was prone to a one‐electron oxidation process for sodium‐ion organic radical batteries.^[^ ^11^ ^]^ And carboxylate‐based compounds 4, functionalized conductive polymers, were subsequently introduced as anodes for OSIBs after inspiration from LIBs.^[^ ^12^, ^13^ ^]^ To date, the development of organic cathode materials suitable for all‐organic full cells remains a significant challenge. Porous organic electrodes 5, which could work as cathodes at p‐dopable potential region and anodes at n‐dopable potential region, have shown promise due to the affordability, recyclability, and high activity for SIBs.^[^ ^14^ ^]^ In 2014, Schiff‐base groups 6 as new redox active sites were explored for SIBs, and the redox potential of Schiff‐based compounds could be adjusted by the proper substitutions.^[^ ^15^ ^]^ Later in 2015, Lei's group found that the extended conjugated systems 7 can strengthen intermolecular interactions and facilitate ion/electron diffusion, significantly influencing subsequent research.^[^ ^16^ ^]^ Beyond these aforementioned materials, additional functional groups have been explored, including the dithiocarboxyl group 8,^[^ ^17^ ^]^ which undergoes reversible three‐electron redox reactions and azo group 9,^[^ ^18^ ^]^ which supports the two‐electron redox process, respectively. These anodes exhibited high capacity and long‐cycle stability, offering new storage categories for SIBs. Those evolving organic materials continuously inspire the development of high‐performance and versatile organic materials for SIBs.

As research progresses, organic materials with multiple active sites deliver high energy density, attracting the researcher's growing interest. Organic molecules, such as disodium rhodizonate (Na_2_C_6_O_6_) cathode with a high theoretical capacity of 501 mAh g^−1^ for reversible four‐electron sodium storage, have gained more attention.^[^ ^19^, ^20^ ^]^ Besides, metal–organic frames (MOFs) and covalent‐organic frames (COFs) become hot points in the past decade, due to their unique periodic structure and rich porosity.^[^ ^21^ ^]^ For instance, 2D conductive MOFs 10 are constructed via conjugated ligands and redox‐active linkers to promote low conductivity and unstable structure, possessing three‐electron storage for each unit.^[^ ^22^ ^]^ Similarly, nitrogen‐rich COFs 11 designed with 12‐electron transfer could narrow down the bandgap between the lowest unoccupied molecular orbital (LUMO) and the highest occupied molecular orbital (HOMO), thus improving ionic/electronic conductivity, and exhibiting a high rate and cycling performance for SIBs.^[^ ^23^ ^]^ Furthermore, organic materials exhibit the versatility to accommodate alkali metal ions with varying ionic radii, suggesting that the exploration of a certain class of organic material can have broader applications across all alkali metal batteries (AMBs).^[^ ^24^ ^]^ This adaptability has driven increasing research interest in broadening organic materials to a wider range of energy storage systems. Recent studies have also highlighted the potential of bipolar small molecular 12, with strong π–π intermolecular forces and poor solubility in multisystem dual‐ion symmetric batteries.^[^ ^25^ ^]^

Tracing the development of organic materials, it is found that most studied organic materials undergo salinization, polymerization, or combination with conductive carbon. This trend arises because many small organic molecules face solubility challenges in organic liquid electrolytes, which is one of the key limitations of organic materials for SIBs. Some organic materials are more stable in solution than in the solid state. Consequently, they tend to dissolve into liquid electrolytes. The solution‐state stability depresses the cycling life and charging and discharging capacities of SIBs, thereby hindering organic materials from competing effectively with inorganic materials. Ultimately, organic materials are considered far from practical for SIBs. Despite the progress of organic materials for SIBs over the past few years, the solubility optimization for OSIBs remains in its early stages. There is a pressing need to systematically explore methods to address this issue and to develop a blueprint for sustainable, long‐lifetime OSIBs.

In this perspective, a systematic discussion is provided on the challenges posed by organic dissolution and its detrimental impact on the performance of SIBs. The discussion considers the roles of electrodes, electrolytes, and separators in addressing these challenges. Building upon this analysis, prevailing strategies for mitigating solubility issues will be summarized through various examples. These strategies span from the electrode and electrolyte to the separator, targeted at improving insolubility and enhancing long‐cycle performance for OSIBs. By leveraging established practices and insights from multisystem batteries, this perspective proposes a forward‐looking guide to provide effective pathways for enhancing insolubility of organic materials for OSIBs toward the achievement of long‐cycling performance.

## 2 Challenges and Strategies to Improve Insolubility for OSIBs

The undesirable dissolution of organic active materials in organic liquid electrolytes remains a significant challenge in the development of long‐cycling OSIBs, which brings about a series of chain reactions that compromise the overall performance of the battery system. From a systemic perspective, the entire battery is generally composed of electrodes, electrolytes, and separators. When organic electrodes are applied in SIBs, some small molecules, such as diamidebenzobisthiazole‐dione (DBTD), dissolve into the electrolytes due to the weak π–π intermolecular interactions, which are insufficient to counteract solvation forces exerted by solvent molecules.^[^ ^26^ ^]^ As a result, these organic active materials fall down from the conductive collector, causing capacity decay after fewer cycles even under a small current density. Some organic solvents such as acetonitrile are found to interact with alkali ions through solvation, and may even undergo reduction, forming organic salt products.^[^ ^27^, ^28^ ^]^ Subsequently, these products intercalate into organic electrodes during the charging and discharging process, possibly damaging the structural integrity of organic materials and even aggravating the performance degradation of the entire battery system. Worse still, some small molecules may pass through the narrow pores of the separators and reach the counter electrode, where they react with the opposing active material. This phenomenon increases the possibility of severe overcharging and the risk of a short circuit. In all, the combined processes including organic molecules dissolution, migration across separators, and subsequent interference with the electrolyte and electrodes ultimately cause the destruction of organic electrodes, and severely decayed and unstable electrochemical performance.

Research has demonstrated that within the first few cycles, the dissolution of organic electrodes can result in a loss of over 90% of the specific capacity, highlighting the critical need for efficient mitigating solubility strategies.^[^ ^29^ ^]^ In terms of experimental research, solving this problem is essential for attaining ultra‐long‐cycle performance in OSIBs and then optimizing the potential of organic materials in SIBs. Practically speaking, the utilization of organic electrode materials in next‐generation energy storage systems would be severely hampered by unresolved instability brought on by material disintegration. To increase the overall effectiveness and financial feasibility of organic electrodes in SIBs, these abovementioned challenges must be overcome.

**Figure 2:**
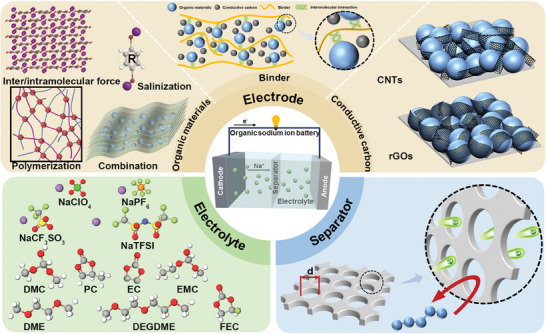
Illustration of strategies to improve insolubility for organic sodium‐ion batteries.

In recent decades, studies have been reported to improve the insolubility of organic materials for OSIBs. As illustrated in **Figure** **2**, these approaches systematically address the solubility challenges of organic compounds in the battery system, focusing on the electrodes, electrolytes, and separators. First, insoluble organic electrode design: the development of insoluble organic electrodes plays a crucial role in mitigating dissolution issues. Key components of electrode materials, including active materials, binders, and conductive additives, are supposed to be deeply analyzed due to their significant and unignorable influence on electrochemical reduction reactions and battery stability. Insoluble organic molecules typically have stronger intermolecular/intramolecular forces against dissolution. For example, organic molecules with large π‐conjugated systems demonstrate superior insolubility in most organic solvents.^[^ ^30^ ^]^ As a result, these organic materials delivered an unexpected electrochemical performance. Additionally, salinization can enhance the polarity of organic hybrid materials, improving stability in lower‐polar nonprotic electrolytes.^[^ ^24^ ^]^ Polymerization is able to enhance the molecular weight to promote stability in various organic solvents.^[^ ^15^ ^]^ Furthermore, the incorporation of conductive networks, such as reduced graphene oxide (rGO) composites and MXenes, can significantly restrict the dissolution of organic materials in electrolytes. Meanwhile, the selection of binder and conductive additives is essential to maintain structural integrity and ensure long‐term cycling performance. Second, electrolytes optimization: studies have revealed that high‐viscosity electrolytes can suppress the dissolution of organic material and form a surface protective layer, thereby improving stability.^[^ ^31^ ^]^ Third, separator modulation: functionalized separators offer a promising solution for preventing dissolved species from shuttling between electrodes. By obstructing the migration of these species, they can minimize the possibility of direct contact between the anode and cathode, thereby reducing the risk of cross‐contamination and performance degradation.^[^ ^32^ ^]^ These strategies, ranging from electrode design to electrolyte optimization and separator modulation, address the dissolution challenges of organic materials in OSIBs. By enhancing the stability and functionality of organic components, and ameliorating other battery components, these approaches contribute to improved efficiency and prolonged lifespan of OSIBs.

## 3 Insoluble Organic Electrode Design

Electrodes consisted of active materials, conductive carbon, and binders in the slurry preparation. Generally, the type of active material determines the energy density; the added conductive additive promotes the electronic conductivity between active materials and the current collector; and binders are used to tighten the connection between the current collector and active materials.^[^ ^33^ ^]^ Organic electrodes are ideally designed to be insoluble in the liquid electrolyte to realize the long‐term cycling performance of OSIBs. The dissolution of organic materials will lead to the loss of active materials, low Coulombic efficiency, and poor cycling stability during the charge and discharge process. In order to promote the insolubility of organic materials, the electrode design is the decisive part of improving electrochemical performance. Strategies referring to active materials, conductive carbon, and binders for promoting insolubility on electrodes are summarized in the following sections.

### 3.1 Active Material

Among the three constituents mentioned above, the active material is the core factor that affects the electrochemical performance of a battery system. A large number of tuneable structures and possible functionalization in organic materials are available for designed high‐energy‐density and power‐density sodium storage.^[^ ^34^, ^35^ ^]^ They have been divided into three categories: n‐type (carbonyl, imine, azo, nitrile, and disulfide), p‐type (amine and thioether), or bipolar‐type (organic radical and conductive polymer) depending on the redox mechanism of organic materials.^[^ ^36^, ^37^ ^]^ However, the organic solubility in the organic electrolyte system causes self‐charging and unstable cycling performance, which will prevent the development of organic materials. It is proven that molecular design and conductive substrate combination can improve the insolubility of organic materials for SIBs.

#### 3.1.1 Molecular Design

As mentioned, high solubility of organic materials in aprotic electrolytes leads to the rapid decay during cycling performance for OSIBs. Fortunately, organic molecules display highly tuneable and controllable properties. This issue can be mitigated by adjusting specific functional groups into the molecular skeletons. Molecular design strategies include strengthening inter/intramolecular interactions, salinization, and polymerization. Such a highly tuneable and designable organic design helps achieve the desirable electrochemical performance for SIBs.

**Figure 3:**
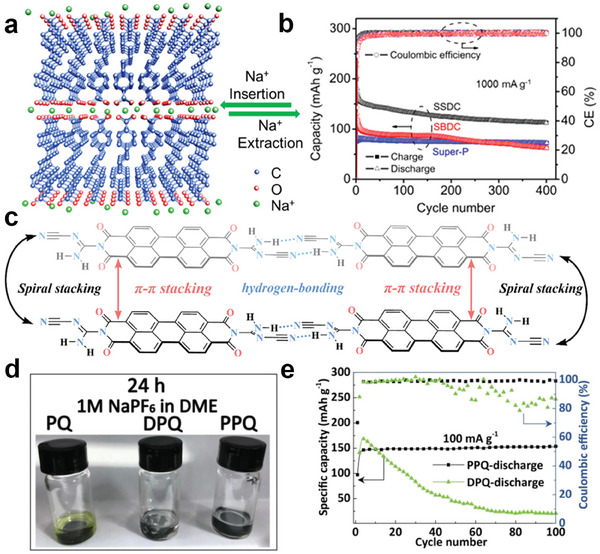
a) Schematic diagram of SSDC with layered stacking via π–π intermolecular interaction. b) The cycling performance of SSDC and the controlled samples at 1 A g^−1^. Reproduced with permission.^[^ ^16^ ^]^ Copyright 2015, American Chemical Society. c) Schematic diagram of P‐DCD stacking mode via hydrogen bonding and π‐conjugation. Reproduced with permission.^[^ ^42^ ^]^ Copyright 2024, Wiley‐VHC GmbH. d) The photograph of the solubility test for phenanthraquinone (PQ), 3,3′‐(1,4‐phenylene)bis(phenanthrene‐9,10‐dione (DPQ), and poly(9,10‐phenanthraquinone‐alt‐benzene) (PPQ)‐based electrodes. e) The cycle performance of DPQ and PPQ at 0.1 A g^−1^. Reproduced with permission.^[^ ^43^ ^]^ Copyright 2022, Elsevier.

Organic materials consist of molecules characterized by weak inter/intramolecular interaction, such as π–π conjugation, hydrogen bonding, and van der Waals forces. These strengthening intermolecular and intramolecular forces are stronger than solvent forces exerted by solvent molecules, thus suppressing the solubility of small molecules in liquid electrolytes.^[^ ^38^ ^]^ Among those strategies, π–π conjugation has emerged as a widely applied approach for both cathodes and anodes in OSIBs. Our previous research demonstrated that the extended π‐conjugated systems in planar organic anodes can enhance intermolecular interaction, thus promoting the hierarchical stacking structure (**Figure** **3**a) and stabilizing cycling performance in sodium storage (Figure 3b).^[^ ^16^ ^]^ Similarly, Fan's group reported that organic cathodes with more than five conjugated aromatic rings exhibited better insoluble in most organic electrolytes.^[^ ^25^, ^39^ ^]^ By extending the conjugation to include 13 and even 16 aromatic rings, they achieved ultralong cycling performance exceeding over 4000 cycles. To date, several planar function groups such as aromatic rings, C≡C, C≡N, and N═N groups have been introduced to extend π‐conjugated systems.^[^ ^40^, ^41^ ^]^ However, despite these advancements, structures based on extension π‐conjugated systems may collapse during the sodiation process due to solvent‐molecule co‐intercalation. This collapse can result in phase separation and dissolution, which limits the long‐term stability of these materials.^[^ ^30^ ^]^

The combination of hydrogen bonding in the organic materials can further strengthen the intermolecular interactions against solvation.^[^ ^44^ ^]^ Functional groups with high electronegative such as ─OH and ─NH_2_ can form hydrogen bonding with the oxygen‐containing redox actives in organic materials.^[^ ^45^, ^46^ ^]^ Consequently, the stronger intermolecular force between molecules can prevent organic dissolution and maintain long‐term cycling performance. Vividly shown in Figure 3c, π–π stacking as well as hydrogen bonding promotes the ordered construction of hydrogen organic frameworks (HOFs), displaying high stability and solubility in most organic solvents.^[^ ^42^ ^]^ The abundant existence of hydrogen bonding in organic materials further enables self‐healing to retain the crystalline structure and suppresses pulverization and dissolution during cycling for OSIBs.^[^ ^47^ ^]^

Salinization, as a kind of molecular design strategy, is aimed at enhancing more stable ionic compounds by introducing Na^+^ ions to replace H atoms in organic materials. This process increases the polarity of organic molecules, effectively preventing organic dissolution in electrolytes and promoting electronic transfer during cycling. So far, carboxylate‐based anodes and alkoxide‐based cathodes are frequently exploited through salinization due to the electrochemical stability of those organic salts, distinct and suitable potential plateaus, and exceptional long‐term cycling performance in SIBs.^[^ ^48^, ^49^ ^]^ It is found that the insolubility of organic salts in aprotic electrolytes can be improved with the increasing valence of anions. However, a major obstacle for organic salts in energy storage applications is that they suffer from considerable solubility in the free state of alkali metal ions during the charging process.^[^ ^50^ ^]^ Therefore, introducing extended π‐conjugated systems or shifting from monomer units to polymers can stabilize the molecular structure. The modifications can solve the above dissolution issue, even if the organic salt materials are in an unstable full‐charge state during desodiation.^[^ ^51^ ^]^

In early research, some insoluble polymer electrodes, especially crosslinked polymers, for energy storage have been studied since the discovery of conductive polymers.^[^ ^52^ ^]^ In general, the monomer units for polymers tend to dissolve into the electrolyte and show severe capacity decay during cycling performance.^[^ ^53^ ^]^ Compared with monomers with the same structure, polymers with extended π‐conjugation can stabilize in liquid electrolyte for 30 days.^[^ ^54^ ^]^ Mostly, polymers with long benzene ring chains exhibit large molecular weights and 3D network structures, which can reduce solubility and promote mechanical strength and structure stability for OSIBs to achieve ultra‐long‐cycling performance.^[^ ^55^, ^56^ ^]^ It is seen in Figure 3d that the small molecular (phenanthraquinone (PQ)) dissolves in the ester‐based electrolyte, while poly(9,10‐phenanthraquinone‐*alt*‐benzene) (PPQ), showing a clear colorless solution, is basically insoluble.^[^ ^43^ ^]^ As expected, polymer electrodes displayed stable cycling performance without attenuation (Figure 3e). However, the purification, degree of polymerization, and synthesis process are still challenges for organic polymers through existing measurements. The following explorations on characterization methods are necessary for in‐depth compression of polymer electrodes in SIBs.

Porous polymers such as COFs and MOFs with enriched porous and ordered structures are applied in energy. Generally, COFs and MOFs are synthesized through the solvothermal method, hydrothermal reaction, and mechanochemical synthesis.^[^ ^57^, ^58^ ^]^ The particle size, phase purity, and morphological architecture of those organic frames are affected by the synthesis method, reaction temperature, reactant concentration, and pH values.^[^ ^59^ ^]^ The earliest research found that organic ligands were completely decomposed in aprotic electrolytes, causing crustal structure collapse during cycling.^[^ ^21^ ^]^ Recently, conjugated monomer units and linkages have been utilized frequently to form conjugated coordination polymers (CCPs).^[^ ^60^, ^61^, ^62^ ^]^ The coordinated bonds can not only immobilize the organic ligands to ensure a robust structure, but also provide new and rich redox active sites.^[^ ^63^ ^]^ These abundant redox sites contribute to high specific capacity, even as high as inorganic materials for sodium storage, which is much more attractive and become a hotspot in recent research. For example, designed COFs with 12 active sites in each repetitive unit maintained 98% capacity retention after thousands of cycles (515 mAh g^−1^ for high theoretical capacity) for sodium storage.^[^ ^23^ ^]^ Importantly, monomer units with extension of π‐conjugated systems can not only improve the insolubility of molecules, but also accelerate electronic transport during desodiation/sodiation for OSIBs.^[^ ^64^ ^]^ For example, triazine, hexaazatriphenylene (HAT), hexaazatrinaphthalene (HATN), octahydroxyltetrabenzoanthracen (TBA), and hexahydroxyltriphenylene (HHTP) monomer units display rich active sites and extended π‐conjugation.^[^ ^65^, ^66^ ^]^ Those synthesized organic frames exhibit minimal capacity decay after thousands of cycles at large current densities.

#### 3.1.2 Conductive Substrate Combination

Another intriguing way to overcome the high solubility is by combining conductive substrates with electrode materials. Conductive substrates possess porous structure and high ionic/electronic including 0D mesoporous carbon, 1D carbon nanotubes (CNTs), 2D rGOs, and layered MXenes.^[^ ^67^ ^]^ The introduction of conductive substrates is feasible to encapsulate small molecules from dissolving in the aprotic electrolytes and improve the entire electronic/ionic conductivity of hybrid organic electrodes.^[^ ^68^ ^]^ Eventually, the long cycling stability and satisfied rate capability of organic composites can be achieved simultaneously for SIBs.

**Figure 4:**
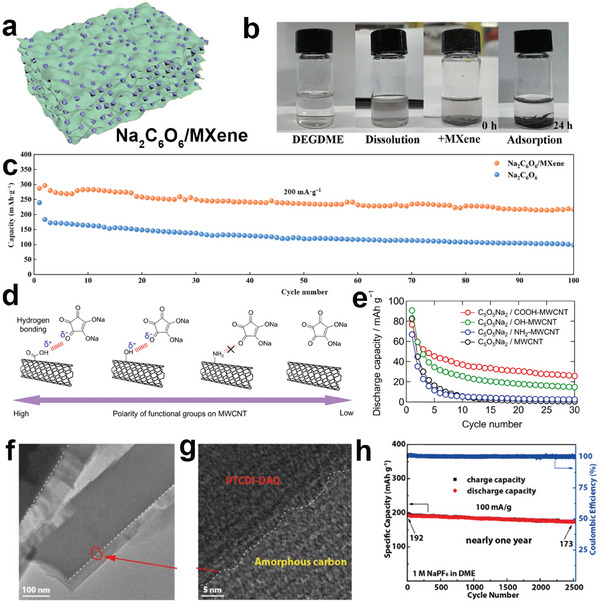
a) The illustrated structure of free‐standing Na_2_C_6_O_6_/Mxene paper. b) Photograph of diethylene glycol dimethyl ether anhydrous (DEGDME) electrolyte, Na_2_C_6_O_6_ dissolving in DEGDME electrolyte, Na_2_C_6_O_6_/MXene dissolving in DEGDME electrolyte for 0 and 24 h. c) Long cycling performance of Na_2_C_6_O_6_/MXene at 0.2 A g^−1^. Reproduced with permission.^[^ ^71^ ^]^ Copyright 2022, Springer Nature. d) Schematic illustration of C_5_O_5_Na_2_ composite with decreasing polarities of these functional groups on MWCNT (COOH─MWCNT, OH─MWCNT, NH_2_─MWCNT, and nonfunctionalized MWCNT). e) The cycling performance of C_5_O_5_Na_2_/COOH─MWCNT, C_5_O_5_Na_2_/OH─MWCNT, C_5_O_5_Na_2_/NH_2_─MWCNT, C_5_O_5_Na_2_/MWCNT for SIBs. Reproduced with permission.^[^ ^75^ ^]^ Copyright 2024, Elsevier. f) TEM and g) HR‐TEM images for PTCDI‐DAQ@C. h) The long‐cycle profile at 100 mA g^−1^. Reproduced with permission.^[^ ^76^ ^]^ Copyright 2023, Wiley‐VHC GmbH.

The top‐down approaches and bottom‐up approaches are applied to construct stable and strong carbon networks for organic materials. Vacuum‐assisted filtration is a simple and feasible top‐down approach to preparing organic–inorganic hybrid composites.^[^ ^69^ ^]^ These uniformly dispersed conductive substrates (MXenes and rGO sheets) combined with organic molecules even can be fabricated into flexible electrodes.^[^ ^70^ ^]^ For example, Na_2_C_6_O_6_ power was dispersed in MXene suspension to form a mixed Na_2_C_6_O_6_/MXene paper by vacuum filtration.^[^ ^71^ ^]^ This layer‐by‐layer structured Na_2_C_6_O_6_/MXene paper (**Figure** **4**a) is stable in the ether electrolytes, indicating that a layered structure can effectively prevent organic molecular dissolution in liquid electrolytes (Figure 4b). Thus, this free‐standing organic composite showed cycling stability as cathodes for SIBs in Figure 4c. Besides, the flexible nanofibers can also be fabricated via encapsulating organic materials into micro/nanotunnels, facilitating Na‐ion diffusion, and restricting organic molecular dissolution.^[^ ^72^ ^]^ Spray drying is a feasible method that can be applied in both water‐soluble and oil‐soluble materials to synthesize organic/inorganic composites.^[^ ^73^ ^]^ Na_2_C_6_H_2_O_4_/CNT nanocomposite was successfully prepared via mixed 2,5‐dihydroxy‐1,4‐benzoquinone mixed with conductive carbon to form homogeneous solution under a spray‐dried process.^[^ ^74^ ^]^ Microsized porous Na_2_C_6_H_2_O_4_/CNT particles were obtained with CNTs wrapped with the sphere surface to form a conductive network, which allows better electronic conductivity. In contrast, the capacity of Na_2_C_6_H_2_O_4_ electrodes severely decayed to 50 mAh g^−1^ only after ten cycles.

Bottom‐up approaches such as hydrogen‐assisted compound and self‐carbonization for conductive substrate combination are more interesting in organic materials’ preparation. Cathode and anode organic materials possess oxygen‐containing functional groups, such as carbonyl, which are able to form hydrogen bonds with conductive substrates terminated by polar functional bonds.^[^ ^77^ ^]^ For example, MXenes with terminal ─OH groups can form hydrogen bonds (O─H…O) with C═O of 1,2,4‐benzenetricarboxylate (TBC) molecules, thus strengthening interactions between MXenes and TBC to prevent molecular dissolution.^[^ ^78^ ^]^ Further investigation found that the strength of hydrogen bonds increases with the enhanced polarity of functional groups on the surface of conductive substrates (COOH─ > OH─ > NH_2_─ ≈ nonfunctionalized in Figure 4d).^[^ ^75^ ^]^ Therefore, the COOH–multiwalled carbon nanotubes (MWCNTs) supported C_5_O_5_Na_2_ cathode exhibited better cycling stability for SIBs (Figure 4e). Due to the composition of C, H, O, etc. in organic material, precise self‐carbonization as a chemical way can transform the surface organics into amorphous carbon layer.^[^ ^76^ ^]^ As shown in Figure 4f,g, the thin carbon layer coats the N,N′‐bis(2‐anthraquinone)‐perylene‐3,4,9,10‐tetracarboxydiimide (PTCDI–DAQ) particles and forms the organic–inorganic core–shell structure. This protective carbon layer can not only suppress the dissolution of organic molecules but also accelerate the ion‐dissolving process, thus maintaining organic structure and promoting ion diffusion. Finally, organic‐carbon core–shell structured PTCDI‐DAQ@C delivered a remarkable cycling performance for SIBs after 2500 cycles (Figure 4h).

Conductive substrate combinations via top‐down and bottom‐up approaches have shown significant influences in preventing anode and cathode organic molecules from dissolving in the liquid electrolytes for SIBs. The stable organic/inorganic composites, especially those connecting with intermolecular force, can improve ion diffusion and structural integrity for the cycle performance for OSIBs. Although it is an effective method to improve solubility issues, the unlimited addition of conductive compounds might reduce the capacity contribution of organic materials and cause the loss of focus on organic active materials. It is also noticeable that the controlled samples of added conductive substrates are necessary to eliminate the impact on capacity contribution clearly.

### 3.2 Conductive Additive and Binder

Most organic materials possess relatively poor electronic conductivity. Although some conductive polymers (e.g., polyaniline (PANI), and polypyrrole (PPy)) display satisfactory conductive properties, the large molecular weight and irreversible reactions prevent the achievement of high energy density and cycling stability.^[^ ^56^ ^]^ Conductive additives with high conductivity are used in a high proportion in the electrode preparation process.^[^ ^79^ ^]^

**Figure 5:**
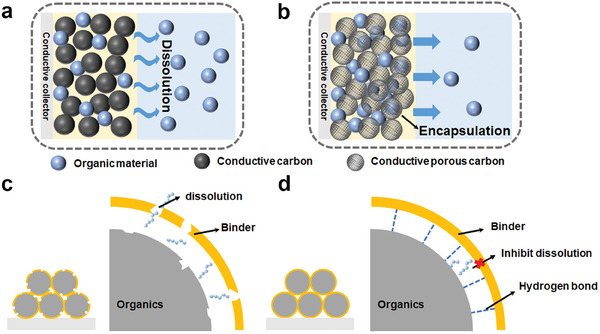
The schematic diagram of a) conductive carbon and b) porous conductive carbon in organic sodium‐ion batteries. Illustration of organic dissolution c) under binder decomposition, and d) under binder protection with hydrogen bonds.

The organic materials dissolve in the electrolyte, then diffuse, transfer across the separator, and reach the surface of anodes driven by the concentration gradient as shown in **Figure** **5**a. These dissolved organics transfer back to the cathodes under the effect of electric field and concentration difference. This phenomenon of the repeated migration of organics between anode and cathodes is known as shuttle effect.^[^ ^80^ ^]^ The research found that conductive additives with rich pores and large specific surface areas like Ketjen black (KB) are capable of accommodating organic materials, reducing organic dissolution (Figure 5b).^[^ ^81^ ^]^ Therefore, multidimensional conductive additives including 0D carbon spheres, 1D CNTs, 2D rGOs, and 3D conductive networks are applied to organic electrodes.^[^ ^82^ ^]^ However, considering that most contribution of specific energy of electrode should come from the active materials, the proportion balance between the conductive carbon and active materials needs to be carefully explored in OSIBs.

Although used in small doses, binders with suitable adhesiveness, thermodynamic stability, and homogeneous dispersibility are critical to maintaining the integral structure of the electrode in the whole battery. They are able to prevent the collapse of electrodes, and the loose contact between the electrode and the current conductor, thus ensuring the continuous charge transfer. Therefore, a desired binder for OSIBs is supposed to have a stronger intermolecular interaction between the active material and the binder. It can keep structural integrity and reduce solubility in organic electrolytes.

Binders applied in battery systems are mainly divided into two categories: organic‐soluble and water‐soluble binders. However, most organic materials tend to be anions after obtaining electrons during the discharging process, some of which may act as nucleophiles. Compared with water‐soluble binders, the degradation of some organic‐based binders accelerates the dissolution of organic materials into the liquid electrolyte (Figure 5c).^[^ ^83^ ^]^ For example, poly(vinylidene fluoride) (PVDF)) binder displays electrical insulation and relatively weak van der Waals forces with organic active materials, which limit the development of PVDF‐based batteries.^[^ ^84^ ^]^ Besides, PVDF, as a fluorine‐containing material, decomposed during cycling as it reacted with alkali to form fluoride ion species.^[^ ^85^ ^]^ Green and sustainable water‐soluble binders such as sodium carboxymethyl cellulose (CMC), beta‐cyclodextrin (beta‐CD), and sodium alginate (SA) are usually applied to form organic electrodes.^[^ ^86^ ^]^ On the one hand, the water‐based binders have good compatibility with the organic electrode material, can provide excellent adhesion to active materials, form a homogeneous slurry, and reduce electrode delamination.^[^ ^87^ ^]^ On the other hand, hydroxyl‐rich aqueous binders such as CMC and SA can form hydrogen bonds with oxygen‐rich functional groups (such as carboxyl or ketone groups) of organic active materials (Figure 5d), thereby effectively preventing dissolution of organic materials, stabilizing electrode structure, and prolonging cycling performance.^[^ ^88^ ^]^ Even for high‐loading organic electrodes (≈10 mg cm^−2^), the water‐based bonder (e.g., polytetrafluoroethylene (PTFE) is still capable of maintaining the integrity of the electrode structure, preventing the organic dissolution and electrode collapse during cycling.^[^ ^89^ ^]^

## 4 Electrolyte Optimization

Although the electrochemical performance of battery systems mostly depends on the electrode, the electrolyte remains one of the unignored factors that affect the performance of the electrode/electrolyte interface, overall battery performance, and safety. Generally, the electrolytes can be divided into three categories: aqueous electrolytes, organic liquid electrolytes, and solid‐state electrolytes. Among them, organic liquid electrolytes stand out as the optimal choice, offering a balanced compromise among safety, cost, and material compatibility.^[^ ^90^ ^]^ The organic electrolytes containing salts, solvents, and additives should possess the necessary advantages: high ionic conductivity and electrochemical stability.^[^ ^91^ ^]^ For OSIBs, the electrolytes are designed to have high ion transport and stable electrochemical properties, thus preventing organic solubility to improve the battery performance of organic materials.

**Figure 6:**
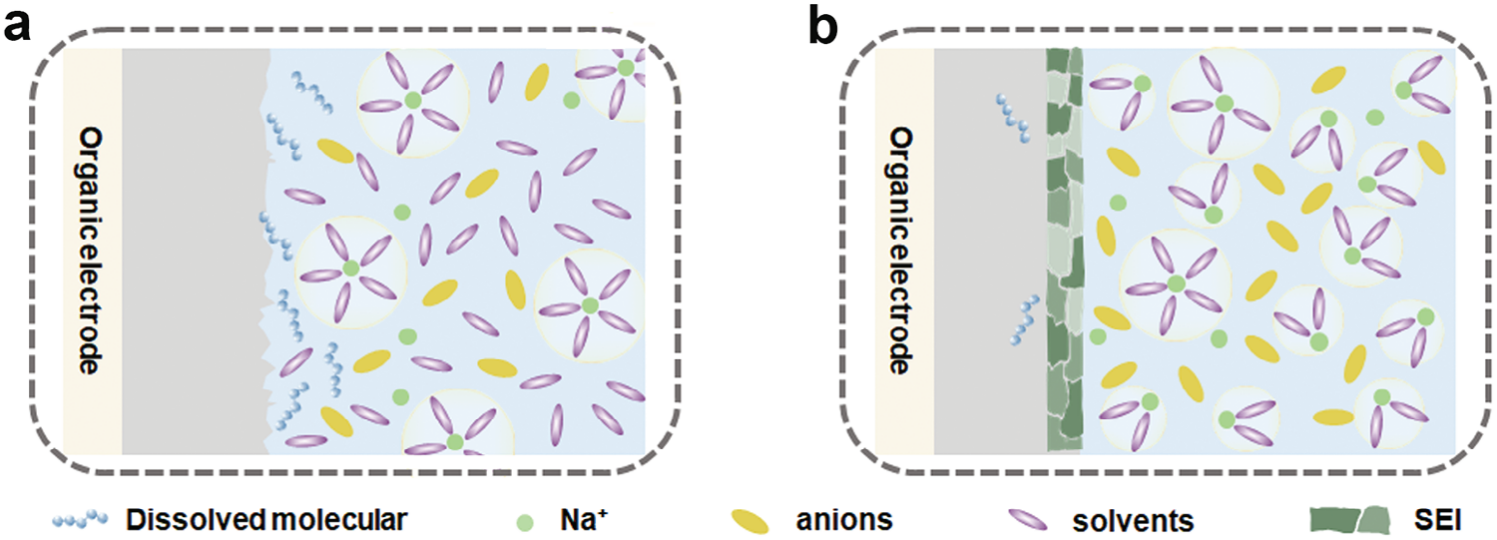
The illustration of Na ions and organic molecules diffusion in a) low‐concentration electrolytes without additives and b) high‐concentration electrolytes with additives.

Metal salts, as the main constituents of electrolytes, have an influence on the ionic conductivity of electrolytes, the composition of solid electrolyte interphase (SEI), and the intercalation/deintercalation reactions with organic materials.^[^ ^92^ ^]^ In low‐concentration electrolytes, organic molecules tend to dissolve due to the more extra solvents in the electrolytes (**Figure** **6**a). In contrast, high‐concentration electrolytes are beneficial for desolvation ability, molecular immobility due to the high viscosity for slow ion/molecular diffusion, and the formation of a stable protective layer (Figure 6b). Elaborately, these high‐concentration electrolytes with less polar solvent molecules can avoid the solvation with Na ions.^[^ ^30^ ^]^ It reduces the possibility of solvent–ion co‐intercalation, maintaining the structural integrity of organic materials with ultralong cycling performance for OSIBs. On the other hand, high‐concentration electrolytes (≥4 m) can increase the viscosity of electrolytes, decreasing the mobility of large dissolved molecules, and thus depressing the dissolution of organic materials.^[^ ^93^ ^]^ Additionally, the added electrolyte additives such as (fluoroethylene carbonate, FEC) for OSIBs with a proportion of 5–10% (according to mass or volume fraction) can promote electrochemical performance via constructing a stable electrode/electrolyte interface, increasing operating voltages, and suppressing aggressive reactions.^[^ ^31^ ^]^ However, there are still some problems with liquid electrolytes that need to be solved. The electrolyte anions such as PF_6_^−^ can be decomposed with labile protons (existing in organic materials) in high‐polarity solvents (propylene carbonate (PC) and ethylene carbonate (EC) ester electrolytes).^[^ ^94^ ^]^ Unlike liquid electrolytes, solid‐state electrolytes represent an alternative approach, with good safety and mechanical strength for OSIBs to prevent the dissolution of organic materials. The solid‐state electrolytes including inorganic‐based, polymer‐based, and organic–inorganic‐mixed solid‐state electrolytes can work as a separator and electrolyte simultaneously.^[^ ^95^ ^]^ Consequently, the solid‐state structure with fewer solvents can reduce organic molecular dissolution effectively, resulting in reversible cycling stability for solid‐state SIBs.^[^ ^96^ ^]^

## 5 Separator Modulation

Separators play a critical role in battery systems and significantly influence battery safety. It is found that the functionalized separators can block the dissolved organic materials from shuttling to the other side, thus promoting cycling stability. Generally, separators can be divided into three types: organic separators, inorganic separators, and organic–inorganic‐hybrid separators.^[^ ^97^ ^]^ On the one hand, the separator can physically prevent the anode and cathode from directly contacting each other. On the other hand, the separator can retain the electrolyte to facilitate ion diffusion inside the battery. Given the critical role of the separator in the battery system, functionalized separators should ideally exhibit enhanced chemical stability, robust mechanical strength, excellent flame retardancy, high electrolyte affinity, stable ionic permeability, and cost‐effectiveness.^[^ ^98^ ^]^ Therefore, separators meeting the above requirements can be developed for safety and long‐lifetime battery systems.

**Figure 7:**
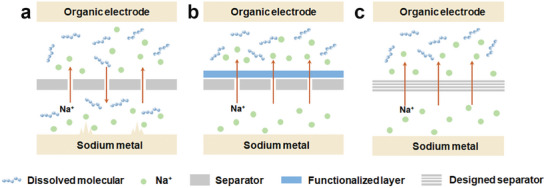
Schematic diagram of Na ions and organic molecules diffusion using a) common separators, b) surface‐modified separators, and c) non‐surface‐modified separators for OSIBs, respectively.

Commonly, commercialized separators possess large pore sizes and poor ionic conductivity, which is insufficient to prevent the shuttle effects of organic solvent and facilitate the orderly conduction of Na ions (**Figure** **7**a). This inadequacy results in unstable sodium deposition, the increasing growth of sodium dendritic, and the direct contact of organic active materials and sodium metal.^[^ ^99^ ^]^ To achieve fast Na ions’ transport and prevent organic materials from shuttling for OSIBs, an artificial protective layer is coated on a commercial separator (surface‐modified separator) to suppress the dissolution of organic molecules upon cycling (Figure 7b). In the early research, rigid structures such as Al_2_O_3_ and carbon spheres as a protective layer were introduced to restrict organic molecules’ diffusion space.^[^ ^100^, ^101^ ^]^ Although the protective layers of different materials and the deposition thickness are manageable to control, these sturdy stacking structures are too densely packed, causing hysteresis in ion migration during the first several cycles.^[^ ^102^ ^]^ Besides, the poor mechanical properties of surface‐modified separators will result in fragility and susceptibility to breakage during synthesis and battery assembly processes, leading to invalidation. Flexible protective films such as polymers and graphene oxide (GO) with high ionic conductivity were applied to inhibit the shuttle effect of organic electrodes.^[^ ^103^, ^104^ ^]^ These separators decorated with flexible films can maintain the integrated structure during assembly. Additionally, the flexible layer is able to suppress organic materials from the shuttle, thus allowing long‐cycling capability and higher Coulombic efficiency for OSIBs. In all, it is prone that an artificial protective layer has an influence on the reduction of organic molecules shuttle. However, the construction of a multilayer and high‐thickness protective membrane will lead to poor interfacial contact and larger charge‐transfer barriers. Therefore, designing a thinner artificial protective layer (microlevel at present) as well as few‐layer or even monolayer separators can meet the requirement for practical development.

Besides, non‐surface‐modified separators with unique intrinsic characteristics are designed to block solvent molecules while allowing the release of Na ions (Figure 7c). These well‐designed separators display exterior‐porous structures or exterior‐nonporous structures. Exterior‐porous separators show rich pores, thus having a good affinity to battery electrolytes. Porous materials such as MOFs and COFs are widely used as separators in battery systems. For example, 2D COFs can adjust the pore size to allow small‐sized cations to pass and limit large‐sized organic molecules to shuttle.^[^ ^105^ ^]^ The π–π stacking COF films display dense pores after orientation deposition under the guideline of rGO. The COF–rGO separator serving as an ionic sieve stabilizes the capacity of soluble pentacenetetrone (PT) after 100 cycles at a large current density (1 A g^−1^). However, a desired separator is fabricated to have a balance among ionic conductivity, selective permeability, dendrite inhibition, and mechanical stability. Nonporous separators with a smooth surface showing fast Na ions’ diffusion give a different strategy to inspire separation preparation for OSIBs.^[^ ^106^ ^]^

## 6 Conclusion and Outlook

SIBs are regarded as a promising complement to LIBs, due to their rich resources, affordability, and safety. Particularly, organic materials have emerged as an ideal possibility for SIB electrode materials, because of their cheap cost, flexibility, lightweight, structural adaptability, and sustainability. These potential active materials, like conductive polymers, carboxylates, Schiff bases, and azo groups, have been steadily developing since the 1980s. However, the solubility issues still limit the achievement of long cycling stability for OSIBs. To guarantee the long‐term and stable application of SIBs, the solubility issue of organic materials must be further resolved in the future through electrode design and battery optimization. We have concluded the advanced long‐cycling performance of insoluble n‐type, p‐type, and bipolar‐type organic materials in the recent 3 years for sodium storage in **Table** **1**. It is vividly shown that the long‐term performance of organic materials is stable even lasting for thousands of cycles, indicating a great potential for the application of organic materials in stabilizing energy output. Therefore, it is essential to give a comprehensive guideline for promoting organic insolubility for SIBs. Our perspective summarizes serious difficulties and provides efficient strategies according to three aspects of electrodes, electrolytes, and separators to address the solubility issue.

**Table 1:** Long‐cycling performance of insoluble organic materials for SIBs in recent 3 years. (organic compounds—pTPA‐AQ: poly‐2,6‐bis(3‐(diphenylamino)phenyl)anthracene‐9,10‐dione, TDT: 2,3,7,8‐tetraamino‐5,10‐dihydrophenazine‐1,4,6,9‐tetraone, CuTP: [5,15‐di(thiophen‐2‐yl) porphinato] Cu(II), NDI‐ONa: N,N′‐dihydroxy naphthalene diimide disodium salt, PAQB: poly(anthraquinone‐alt‐benzene), PNAI‐NGR: multi‐functionalized polymers‐nitrogen‐doped graphene, QAP: quinone‐fused aza‐phenazines, PPDA: p‐phenylenediamine, TABQ: tetramino‐benzoquinone, CPTA: conjugated poly(2,4,6‐tri(thiophen‐2‐yl)‐1,3,5‐triazin, NiTTP: [5,10,15,20‐tetrathienylporphinato] Ni(II), p‐PADA: polymer terephthalaldehyde‐4,4’‐diazoaniline, TTHP‐T: 2,4,6‐tris(2,4,6‐trihydroxyphenyl)‐1,3,5‐triazine, Na‐DCA: 2,2′‐bypyridine‐4,4′‐dicarboxylic acid disodium salt, DQPZ‐3PXZ: diquinoxalino[2,3‐a:2′,3′‐c]phenazine‐2,6,10‐tris(phenoxazine), PQPZ: polymer containing phenanthraquinone and dihy‐drophenazine groups, TPAD: 1,4,5,8‐tetrakis(phenylamino)anthracene‐9,10‐dione, ABPZ: polymer containing dihydrophenazine and azobenzene groups, PNT: polymer polycondensed from N,N,N′,N′‐tetrakis(4‐aminophenyl)‐1,4 phenylenediamine (TAP) and naphthalenete tracarboxylic dianhydride (NTCDA). Conductive additives—AB: acetylene black, CB: carbon black, KB: Ketjen black, GO: graphene oxide, and MWCNTs: multiwalled carbon nanotubes; binders—PVDF: poly(vinylidene fluoride), CMC: sodium carboxymethyl cellulose, and PANa: sodium polyacrylate; electrolytes—EC: ethylene carbonate, PC: propylene carbonate, DMC: dimethyl carbonate, DME: 1,2‐dimethoxyethane, DIGLYME: diethylene glycol dimethyl ether, DEGDME: diethylene glycol dimethyl ether anhydrous, G2: glycol dimethyl ether, TEGDME: tetraethylene glycol dimethyl ether and FEC: fluoroethylene carbonate).

Type of organic materials	Organic compound	Slurry ratio^a)^	Electrolyte	Cycling performance			Year (Refs.)
				Capacity [mAh g^−1^]	Cycles [*n*]	Current density [A g^−1^]	
p‐Type	pTPA–AQ	5:4 (MWCNTs):1 (PVDF)	1 m NaPF_6_ in PC	72	3000	5	2024^[^ ^107^ ^]^
	TDT	7:2 (Super P):1 (CMC)	1 m NaPF_6_ in DIGLYME	184	3500	3	2024^[^ ^45^ ^]^
	PTCDI‐DAQ@C	6:3 (KB):1 (La133)	1 m NaPF_6_ in DME	131	1000	3	2024^[^ ^75^ ^]^
	CuTP	8:1 (AB):1 (PVDF)	1 m NaClO_4_ in PC with 5 wt% FEC	82	11 000	5	2023^[^ ^108^ ^]^
	NDI‐ONa	6:3 (Super P):1 (SA)	1 m NaPF_6_ in DEGDME	≈160	20 000	3	2023^[^ ^87^ ^]^
	NiQAP	4:5 (AB):1 (PVDF)	1 m NaPF_6_ in DME	100.1	10 000	1	2023^[^ ^63^ ^]^
	MXene@PTCDA	3:1 (Super P):1 (PVDF)	1 m NaPF_6_ in EC/DEC with 5 wt% FEC	≈60	1500	0.5	2022^[^ ^109^ ^]^
	PAQB	6:3 (KB):1 (La133)	1 m NaPF_6_ in DME	130	3000	2	2022^[^ ^110^ ^]^
	PNAI‐NGR	6:3 (CB):1 (SA)	1 m NaPF_6_ in DEGDME	100	1200	1	2022^[^ ^111^ ^]^
	PPQ	6:2 (KB):1 (CNTs):1 (La133)	1 m NaPF_6_ in DME	84	700	0.8	2022^[^ ^43^ ^]^
n‐Type	HAT‐CN	8:1 (KB):1 (PVDF)	4 m NaPF_6_ in DME	215	5000	10	2024^[^ ^41^ ^]^
	QAP	5:4 (Super P):1 (PVDF)	1 m NaPF_6_ in DME	421	500	1	2024^[^ ^112^ ^]^
	HAT‐PPDA	5:4 (Super P):1 (CMC)	1 m NaPF_6_ in DME	222	2000	1	2023^[^ ^113^ ^]^
	HHTP‐TABQ	5:4 (CNTs):1 (PVDF)	1 m NaClO_4_ in EC/DMC with 5 wt% FEC	202	1000	5	2023^[^ ^114^ ^]^
	CPTA	6:3 (CB):1 (PVDF)	1 m NaCF_3_SO_3_ in DIGLYME	401	1000	2	2023^[^ ^115^ ^]^
	NiTTP	7:2 (AB):1 (PVDF)	1 m NaPF_6_ in DME	108	1100	1	2023^[^ ^116^ ^]^
	TBC/Ti3C2*T_x_*	8:1 (AB):1 (PVDF)	1 m NaPF_6_ in DME	133	1600	1	2023^[^ ^78^ ^]^
	p‐PADA	5:4 (KB):1 (PVDF)	1 m NaPF_6_ in DIGLYME	313	1000	5	2022^[^ ^54^ ^]^
	TTHP‐T	3:6 (GO):1 (PVDF)	1 m NaPF_6_ in DME	497	1000	1	2022^[^ ^46^ ^]^
	Na_4_PTC	7:2 (Super P):1 (PANa)	1 m NaPF_6_ in G2	110	20 000	2	2022^[^ ^117^ ^]^
	Na‐DCA‐NrGO	8:1 (CB):1 (SA)	1.2 m NaClO_4_ in EC/DEC	126	500	0.2	2022^[^ ^118^ ^]^
Bipolar	DQPZ‐3PXZ	6:3 (KB):1 (La133)	2 m NaPF_6_ in DEGDME	193	4000	1	2024^[^ ^25^ ^]^
	PQPZ	6:3 (KB):1 (PVDF)	1 m NaPF_6_ in DIGLYME	96	10 000	10 C (1 C = 277 mAh g^−1^)	2024^[^ ^119^ ^]^
	TPAD‐COF	6:3 (AB):1 (PVDF)	1 m NaPF_6_ in DME	75	2000	1	2024^[^ ^120^ ^]^
	ABPZ	13:4 (KB):3 (PVDF)	1 m NaClO_4_ in TEGDME	146	1000	8 C	2023^[^ ^53^ ^]^
	PNT	6:3 (CB):1 (SA)	1 m NaPF_6_ in EC/PC	100	200	0.05	2023^[^ ^121^ ^]^

^a)^ The ratio of active material, conductive additive, and binder.

For electrodes, molecular design is a fundamental solution to the solubility problem. Creating extensive π‐conjugated systems, increasing intramolecular and intermolecular forces, salinization, and polymerization can improve the insolubility of organic materials in electrolytes. Besides, establishing conductive networks with organic materials can minimize material dissolution and improve conductivity. Additionally, binder optimization strengthens the electrode structure's integrity, and carbon additives help encapsulate dissolved organics. For electrolytes, high‐concentration electrolytes are employed in battery optimization to prevent organic material dissolution and create a protective coating on the surface. For separators, functionalization can regulate the separator properties and prevent short circuits and undesirable reactions. These strategies intend to enhance stability and long‐term performance for OSIBs.

Meanwhile, the modification for insoluble organic materials in the battery system faces other challenges, including the lower theoretical specific capacity and low mass loading for organic electrode design, slow electronic/ionic transfer for concentrated electrolyte optimization, and transfer pore size for functional separator modulation. Therefore, it is necessary to further investigate organic materials that can resolve the solubility issue and achieve high energy density at the same time in academia and industry. Functional group introduction brings about more active sites in organic materials allowing high theoretical organic materials for SIBs. Exploration of high‐conductive active material helps increase the proportion of active materials in slurry preparation, and additionally accelerates electronic transfer during cycling. Besides, investigating fast ionic diffusion inside organic active materials and across electrolytes and separators allows us to improve sluggish reactions.

## Conflict of Interest

The authors declare no conflict of interest.
